# Simulation-Based Clarification of Appropriate Factors for Presenting Phosphene in Two Directions Avoiding Electrical Interference

**DOI:** 10.3390/bioengineering8080111

**Published:** 2021-08-05

**Authors:** Manami Kanamaru, Phan Xuan Tan, Eiji Kamioka

**Affiliations:** Graduate School of Engineering and Science, Shibaura Institute of Technology, Tokyo 135-8548, Japan; tanpx@shibaura-it.ac.jp

**Keywords:** phosphene, walking support system for blind people, electric field, finite element method, eyeball surface

## Abstract

Walking support systems are essential for blind people. In this study, the presentation of phosphene position is focused on as a method to detect obstacles for blind people. When the phosphene is used in a walking support system, it is necessary to accurately present the phosphene in at least three directions of the visual field. Controlling the presentation of phosphene position has been reported in several previous studies. However, methodologies to present phosphene in multiple directions without any electric interference have not as yet been investigated. In this study, therefore, appropriate stimulation factors are clarified by the simulation of electric field on the eyeball surface which is strongly related to the presentation of phosphene position in the visual field. As a result of the simulation, it was revealed that the distance of each electrode does not give a significant effect to the eyeball surface. However, the phase of alternating current significantly changed the electric field on the eyeball surface. From investigation of the simulation results, it was clarified that the transition of the electric field on the eyeball surface can be controlled using anti-phase stimulation. In addition, the methodology to present the phosphene at least in two directions was verified.

## 1. Introduction

Phosphene is critical visual information for hands-free walking support systems. Basically, it is a flash which is perceived by giving a magnetic or electrical stimulus to the visual pathway [1,2]. Therefore, the phosphene can indicate the obstacle position with visual information as a walking support system to blind people without interference of the other senses. There are different methods for producing phosphene, such as, TMS (trans magnetic stimulation) [3], TES (transcorneal electric stimulation) [4], and tACS (transcranial alternating current stimulation) [5]. However, tACS becomes the most appropriate approach for some reasons. First, no severe side effects have been reported when tACS was used [2,6,7]. Second, the electrode’s placement for tACS can be easily adjusted. TMS needs large equipment for use and the coil placement cannot be adjusted for stimulus while walking [3]. The electrode placement and the phosphene presentation position with TES cannot be changed because the TES electrode is on the cornea of a human eyeball [4]. When phosphene is used with a walking support system for blind people, it is necessary to accurately present the phosphene in at least three directions of vision. Three directions indication of obstacles has been used in previous walking support systems for blind people [8,9]. The reason why the position information of obstacles on the left and right direction are required is that it is necessary to recognize the distance to the obstacle adjacent to the body and maintain a certain distance for their safety [8].

Several previous studies have reported how to control the presentation position of phosphene [10]. Higuchi et al., reported the presentation position of phosphene when electrodes were placed around the eyeball [10]. Their report revealed that phosphene was observed around the electrodes. When the electrodes were placed in the lower part of an eyeball, phosphene was also observed in the lower part of the visual field. Therefore, it is considered that the presentation position of phosphene can be controlled by adjusting the electrode’s placement around eyeballs in consideration of the stimulation position of the eyeballs. However, the current path around the eyeballs while phosphenes are occurring has not as yet been clarified in their study [10].

Laakso et al. [11] reported that the electric current flowed around the eye orbit and directly stimulated the retina. In this paper, this hypothesis is called “conventional theory”. According to their explanation, the nasal current of the eye stimulates the nasal retina, and the current on the temporal side stimulates the temporal retina directly from the outer layer of the eyeball. However, the position of the phosphene presented by Higuchi et al., contradicted this conventional theory [10]. Based on the conventional theory, the current from the electrode placed around the lower part of the eyeball stimulates the lower retina. The lower retina is the area processing the upper part of the human visual field [12], and the phosphene should be observed in the upper part of the human visual field; however, the phosphene was observed in the lower part of the human visual field in the study by Higuchi et al. [10]. Therefore, the conventional theory is inconsistent with the mechanism which explains the spatial relation between retinae and human visual field.

A previous study investigated the current path around the eyeball to explain the presentation position of phosphene without any contradiction. Manami et al. [13] indicated that the visual retina could be stimulated directly when the visual retina is exposed on the facial surface side due to eye movement. Note that the visual retina is not exposed on the facial surface side when the eyeball is gazing forward [13]. In the previous study [13], a new hypothesis has posited that the electric current path around the eyeball directly stimulates only the region exposed on the facial surface side without flowing to the eye orbit as in the conventional theory. In addition, the position of the phosphene with eye movement was mentioned based on the assumption that the retina inside the eyeball was indirectly stimulated by the change in the voltage on the eyeball surface. According to the conventional theory, the electric current around the eyeball stimulates the retina that was not exposed on the facial surface side [10]. However, when the right eyeball is stimulated from the temporal side, phosphene was observed on the right side of the visual field. Moreover, the phosphene was observed on the left side of the visual field only when the temporal retina of the right eyeball is exposed by eye movement [13]. From their observation result, it was shown that the electric current around the eyeball does not flow along the eye orbit or directly stimulate the retina. This new hypothesis is considered to be valid because it does not contradict the presentation position of the phosphene observed in the related study by Higuchi et al. [10]. Therefore, in this study, we confidently assume that the electric current around the eyeball stimulates only the region exposed on the facial surface side, and it is considered that the voltage change on the eyeball surface affects the presentation position of the phosphene.

As mentioned above, towards realizing a walking support system using phosphene, it is indispensable to present a phosphene in at least three directions. From a previous study [13], the phosphene can be presented at the intended position by stimulating the intended region on the eyeball surface. In addition, it is clear from previous studies [10,13] that the placement of the electrodes is important for presenting a phosphene at the intended position. The method of presenting a phosphene in three directions, right, forward, and left, which was reported in the previous study [14], also stimulates the intended region of the eyeball surface by appropriate electrode placement. However, their method does not discuss the case where the phosphene is presented in three directions, right, forward, and left, simultaneously. Hence, it cannot be handled when the obstacles are in two or more directions, right, forward, and left. Since the area of the eyeball surface is limited, electrical interference should be of concern while presenting a phosphene in two or more directions simultaneously. When a large current intensity is used, it may stimulate not only the area on the eyeball surface near the electrodes but also the area around it. Therefore, when two or more pairs of electrodes are used simultaneously, not only the area near each electrode but also the area amplified by overlapping the stimuli from each electrode may be strongly stimulated. In this study, the phenomenon that the unintended region is stimulated by using two pairs of electrodes is considered as electrical interference. When electrodes are placed close to each other, the phosphene might be shown in an unintended direction depending on the stimulation parameters. Therefore, in this study, it is hypothesized that it is possible to stimulate the intended region on the eyeball surface by selecting appropriate factors (electrode placement, current intensity, phase) to present phosphene in two directions simultaneously. Following the previous research [14], the change in the electric field on the eyeball surface under each factor will be verified by simulation, and the factors under which the influence of electrical interference appears will be shown in this paper.

The rest of the paper is organized as follows: In Section 2, the background knowledge is stated. Section 3 indicates the simulation condition of the electric field on the eyeball surface. The simulation result is shown in Section 4, and the method for presenting the phosphene in two directions at the same time is discussed in Section 5. Finally, Section 6 concludes this paper.

## 2. Background Knowledge

In this section, related studies on stimulus factors and electrical interference that could be involved in the presentation of phosphenes are mentioned. Moreover, the relation between electrodes’ placement and the position of phosphenes presentation is discussed based on previous studies.

### 2.1. Stimulus Factors for Human Head

Firstly, electric current intensity as a stimulus is focused on. When applying electrical stimulus to a human’s head, it is necessary to consider the magnitude of the current intensity as a stimulus factor. Especially, the side effects such as pain and itch caused by the electrical current stimulus should be taken into account ethically [3,5,6,7]. However, there is another perspective. The target area in the brain must be selectively stimulated. When an invasive stimulation method is used, it can locally stimulate the target cells easily. However, when a non-invasive stimulation method such as tACS is used, it is necessary to consider the stimulation to cells outside the target area. This is because it may cause unintended effects.

Tan et al., simulated how the electric field value on the cortex changes due to the change of the current intensity during brain stimulation by tACS [15]. Using two pairs of electrodes placed on the pre-supplementary motor area (pre-SMA) and the right inferior frontal gyrus (rIFG), the difference of the effects on the cortex depending on the current intensity of the pre-SMA electrodes was reported. The current intensity of the pre-SMA was varied, which were 1 mA, 1.6 mA, and 2 mA, and the current intensity of the rIFG was fixed at 1 mA, showing the electric field strength on the cortical surface and the affected areas inside the cortex. In the simulation by Tan et al., when both pre-SMA and rIFG were stimulated with 1 mA, the average electric field strength of the pre-SMA was as small as 0.040 V/m, whereas the one of the rIFG was 0.102 V/m, which is fairly larger than the pre-SMA. This difference could be explained by the difference in a skull’s thickness depending on the region of the head [16]. In addition, when the pre-SMA was stimulated with 1.6 mA, the average electric field strength of the pre-SMA was 0.060 V/m, whereas the one of the rIFG was 0.109 V/m, which is much larger than the pre-SMA due to the electric interference by the pre-SMA. When the pre-SMA was stimulated with 2 mA, the average electric field strength of the pre-SMA was 0.073 V/m, whereas the one of the rIFG was 0.114 V/m. It revealed that the magnitude of the electric field strength by the pre-SMA electrode can overcome the inhibition due to the skull’s thickness to some extent by adjusting the current intensity, and can stimulate the target cortex. Furthermore, in their simulation, the stimulated area inside of the cortex was also discussed. According to their results, the higher current intensity at the pre-SMA than that of the rIFG strongly stimulated other regions, which indicates that local stimulation is difficult. Therefore, when stimulating with multiple electrodes, it is necessary to keep in mind the electric interference of current and to consider the trade-off between the electric field strength and the area of stimulation.

It is considered that the electrode placements that present phosphene to an intended direction in the previous study, which was mentioned in Section 1 [14], were arranged closer compared to the distance between rIFG and pre-SMA. As stated above, therefore, stimulation to unintended areas could happen by electrical interference. This is a significant issue to be solved in order to present phosphene at an intended position.

Next, the phase of the stimulation current is focused on. In the case of electrical stimulation to the brain, the purpose of the stimulation is to intervene in neurotransmission. Therefore, the selection of the phase of alternating current has been discussed [17,18].

Ivan et al., varied the phase of the stimulation current from 0 to 360° in 15° steps and reported the magnitude of the electric field in the whole brain with each phase condition [18]. Electrodes were installed on the frontal and occipital regions, and a return electrode was installed on the temporal region. Two types of non-human primates (11-year-old capuchin monkey and 6-year-old rhesus monkey) were utilized to measure their intracranial electric characteristics, evaluating the distribution of voltage and the electric field between the selected electrodes with a stimulation value of 0.1 mA and a frequency of 10 Hz. In the evaluation, 29 and 22 recording electrodes were placed and the results were analyzed from the left occipital gyri to the orbital gyri and from the occipital gyri to the temporal pole, respectively. As a result, both the electric field and the voltage at the recording electrode showed a non-linear increase from 0 to 180° and a non-linear decrease from 180 to 360°. Moreover, the direction of the electric field at each recording electrode position was also reported. In the case of 0°, opposite electric fields were generated in the front and occipital of the head, whereas in the case of 180°, the directions of the electric fields did not change between the front and occipital of the head.

In this study, the presentation of phosphene by eye stimulation is focused on, and it is not possible to directly compare this with the response to the electric field in the brain due to the phase change reported by Ivan et al. [18]. However, it was found that the electric field of the human body changes depending on the phase of the alternating current stimulation, despite the electrodes arranged at a distance such as the frontal region and the occipital region. In the previous study described in Section 1 [14], all the electrodes were placed on the surface of the face during eye stimulation, so that when a multiple electrode is used, it is necessary to consider the change in stimulation to the eye due to the phase difference.

### 2.2. Phosphene Presentation

In this subsection, the relation between electrodes’ placement on the facial surface and the presentation position of phosphene is focused on. As mentioned in Section 1, when the phosphene is being generated, the electric current around the eyeball directly stimulates only the area exposed on the facial surface. Here, the relation between the electrode’s placement and the presented positions of the phosphenes, and the reason why the phosphenes were presented at the positions mentioned in the previous studies are explained.

Six electrode placements were used in the previous study [14]. The forehead part was divided into six areas and one electrode was placed on one of the six areas, and the other electrode was placed on the cheek [14]. When stimulating the right eyeball, the electrodes were placed on the right side of the face, and when stimulating the left eyeball, the electrodes were placed on the left side of the face. When the person is looking to the right-hand side by moving their eyeballs, the nasal retina of the right eyeball and the temporal retina of the left eyeball are exposed on the facial surface, and both of these areas process the right side of the visual field. On the other hand, when the person is looking to the left-hand side by moving their eyeballs, the temporal retina of the right eyeball and the nasal retina of the left eyeball are exposed on the facial surface, and both of these areas process the left side of the visual field [12]. Therefore, regardless of the electrode’s placement, the phosphene was observed on the right side of the visual field when the eyeballs were moving to the right direction. In the same way, the phosphene was observed on the left side of the visual field when the eyeballs were moving to the left direction.

**Figure 1:**
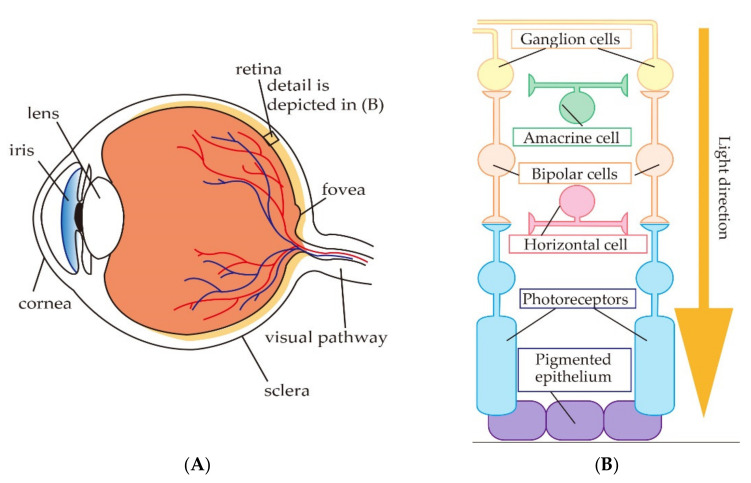
Internal structure of human eyeball: (**A**) Inside of the eye; (**B**) the organization of the retina.

On the other hand, when gazing in the center direction, the visual retina is not exposed on the facial surface, hence, the visual retina is not directly stimulated. An indirect stimulation is presumed to explain the presentation of the phosphene in this case. Another related study has reported the presentation position of phosphene when stimulating the cornea with a coil-embedded contact lens [19]. According to their report, the phosphene was observed on the central visual field. Figure 1A shows the inside of an eyeball, and the central visual field is processed around the fovea. As shown in Figure 1A, the fovea is located on the opposite side of the cornea inside eyeball. Therefore, it can be inferred that electric stimulus around eyeball is possible to indirectly stimulate the retina, which processes the central visual field.

Moreover, from the report by Mihashi et al., it is clear that the electrical stimulus to the cornea does not flow to the eye orbit and does not stimulate the eyeball from the outer layer of the retina [20,21]. Injection of TTX (tetrodotoxin) into the eyeball inhibits the functions of ganglion cells and amacrine cells. Mihashi et al., injected it into the cat eyeball, applied light stimulus and electrical stimulus, and reported the activity of retinal cells. Figure 1B shows the organization of the retina. As shown in Figure 1B, the photoreceptors are located on the outer layer in the retina, and the ganglion cells and amacrine cells are located on the inner layer in the retina. Their report has clarified that the photoreceptors located in the outer layer of the retina did not respond to the electrical stimulus when the functions of ganglion cells and amacrine cells, which are the inner layers of the retina, were inhibited. On the other hand, it was reported that the photoreceptors responded to light stimulus correctly. From their report, the electric current around the eyeball does not flow to the eye orbit and does not stimulate the retina from the outer layer. In addition, when gazing at the center of the visual field, it can be inferred that the electric current indirectly stimulates the inner layer of the retina located on the opposite side of the stimulating area.

Therefore, it is considered that when the temporal area of the right eyeball surface is stimulated, the nasal retina of the right eye is stimulated indirectly, and the phosphene is observed on the right side of the visual field. Similarly, when the vicinity of the central cornea of the right eyeball is stimulated, the vicinity of the macula is stimulated, and the phosphene is observed on the central visual field. In the case of the left eyeball, it is considered that the nasal retina is stimulated when the temporal area of the eye is stimulated, and the phosphene is observed on the left side of the visual field. Similarly, when the vicinity of the central cornea of the left eyeball is stimulated, the vicinity of the macula is stimulated, and the phosphene is observed on the central visual field. In other words, the phosphene can possibly present to an intended area in the visual field by stimulating the nasal side, the central side, and the temporal side of the eyeball surface selectively.

Therefore, it is considered that controlling the presentation of phosphene in two or more directions of the visual field is possible by clarifying how to stimulate the intended regions on the eyeball surface while avoiding the electrical interference.

### 2.3. Simulation Tool of the Electric Field for Human Head Model

This subsection describes the simulation tool of the electric field, which was used in the previous study [14].

An open-source software package for simulating non-invasive brain stimulation, namely, SimNIBS [22,23,24,25], was used in the previous study [14]. FEM (finite element method calculations) [26] are used to obtain the electric field inside the human head in SimNIBS. FEM are one of the numerical calculation methods for solving differential equations based on boundary conditions. It was developed for the analysis of complex structures [26]. The structure handled by SimNIBS is a human head model, which has a highly complex shape. FEM discretizes the head into small sections with simple geometric shapes called elements in modeling this human head. SimNIBS uses a tetrahedron as the elements, however, there are some methods that analyze using other shapes such as a hexahedron [25]. Each element does not overlap, and the apex of each element is shared with some other elements. In FEM, a discretized structure called a domain (in SimNIBS, the head) is called a mesh. Moreover, during the simulation, the electrodes are automatically meshed and handled on the surface of the scalp of the head model. The simulation for phosphene stimulation using TMS and tDCS (transcranial direct current stimulation) is performed by default.

The simulation of tDCS in SimNIBS follows Laplace’s equation in determining the potential [25].

(1) $$\nabla \cdot \left(\sigma \nabla \varphi \right)=0\;\left(in\;\Omega \right),$$

(2) $$E=-\nabla \varphi \;\left(in\;\Omega \right),$$

In Equation (1), σ, φ and Ω indicate the conductivity, the potential caused by an external stimulus, and the region covered by this numerical calculation, respectively. E in Equation (2) indicates the electric field determined by the gradient of the potential. Regarding the boundary conditions in SimNIBS, these are set for the electrode part and other areas. Under the initial conditions, the Dirichlet boundary condition is applied in the electrode part, and the Neumann boundary condition is applied in other areas. In the low-frequency stimulus condition, tACS simulation can be performed by the same method as tDCS simulation. When the simulation is performed by assuming a low-frequency alternating current of 10 Hz, which is the same as the previous study [13,14], the simulation is performed by the same method as tDCS. Additionally, SimNIBS provides a multichannel simulation. In the multichannel simulation with SimNIBS, each electric field created by the electric currents flowing through the electrodes is scaled and summed. In the previous study [14], SimNIBS was used for the simulation with single pair of stimulation, however, SimNIBS can be used for the stimulation with individual two or more pairs of electrodes. Therefore, it can be considered that SimNIBS is also a suitable simulation tool for this study.

## 3. Evaluation Method for Avoiding the Electric Interference While Presenting Phosphene in Two Directions

In this section, based on the background knowledge described in Section 2, a hypothesis regarding the method of presenting a phosphene at two intended positions is described. It also mentions the simulation conditions for testing that hypothesis.

According to [15], electric interference will occur when a stimulus with a large current is applied to the human brain. It can be predicted that this phenomenon will be caused even during eye stimulation, and electrical interference can be avoided by selecting an appropriate current intensity. In addition, previous studies [18] showed that the direction of the electric field between the electrodes changed during brain stimulation with alternating currents of different phases. It is presumed that amplification and cancellation of the current intensity occur by applying electrical stimuli with different phases. Therefore, only the intended region on the eyeball surface can be stimulated by selecting an appropriate phase. Furthermore, as mentioned in Section 1, the electrode position is directly related to the presentation position of the phosphene [10,14]. Therefore, in this study, the hypothesis is posited that the phosphene can be presented at two intended positions by selecting appropriate factors, including the electrode placement, current intensity, and phase.

In this study, the change of the electric field value is simulated while changing each factor (electrode placement, current intensity, phase) is changed, focusing on the transition of the electric field value on the eyeball surface. The simulation of the electric field value on the eyeball surface is performed based on the following two steps.

- Verify how much electrical interference due to the distance between the electrodes affects the transition of the electric field on the eyeball surface. The electrode placements are designed to gradually make the distance between the electrodes larger.
- Clarify how to simultaneously present the phosphene in two directions of the visual field while avoiding electrical interference. The electrode placements that stimulate intended regions on the eyeball surface reported in the previous study are applied in the simulation.

### 3.1. The Simulation Conditions

This subsection describes the simulation condition used in the simulation tool, SimNIBS [22,23,24,25]. In this study, SimNIBS is used to obtain the electric field value on the eyeball surface stimulated by tACS. Therefore, the head model, electrode placements, and stimulation values will be described in this subsection.

The head model used in this study is “Ernie”, which is a sample data set of SimNIBS utilized for the electric field simulation. Ernie is a young and healthy man and his written informed consent to publish MR (magnetic resonance) datasets has been obtained. Moreover, Ernie’s structural MR images have been confirmed by a radiologist and are completely anonymized. Ernie’s face was replaced by the average of the datasets of several other subjects obtained in the same MR sequence. The subject’s dataset consists of high-resolution T1 and T2-weighted images and diffusion MR images. The voxel size in high-resolution T1 and T2-weighted images is 1 × 1 × 1 $m^{3}$, and the voxel size in diffusion MR images is 2 × 2 × 2 $m^{3}$. In addition, the electrical conductivity of body tissues and fluids is assumed to be linear and isotropic. Table 1 shows the electrical conductivity values to electrical stimulation at 10 Hz [27,28] since the frequency value of tACS used our simulation is considered as 10 Hz following those of a previous study [13,14].

**Figure 2:**
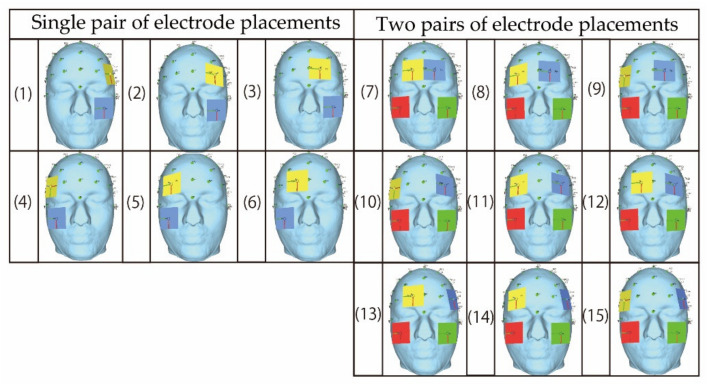
The electrodes’ placement that is gradually separated from each other.

**Figure 3:**
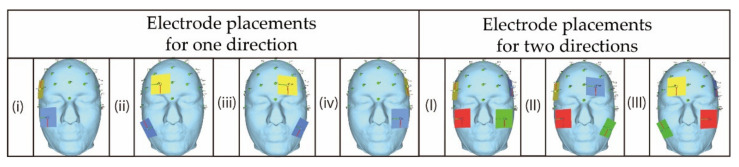
The electrode placement that stimulates the intended region on the eyeball surface [14]; the figures were produced by the authors in SimNIBS (accessed on 2 April 2021).

**Figure 4:**
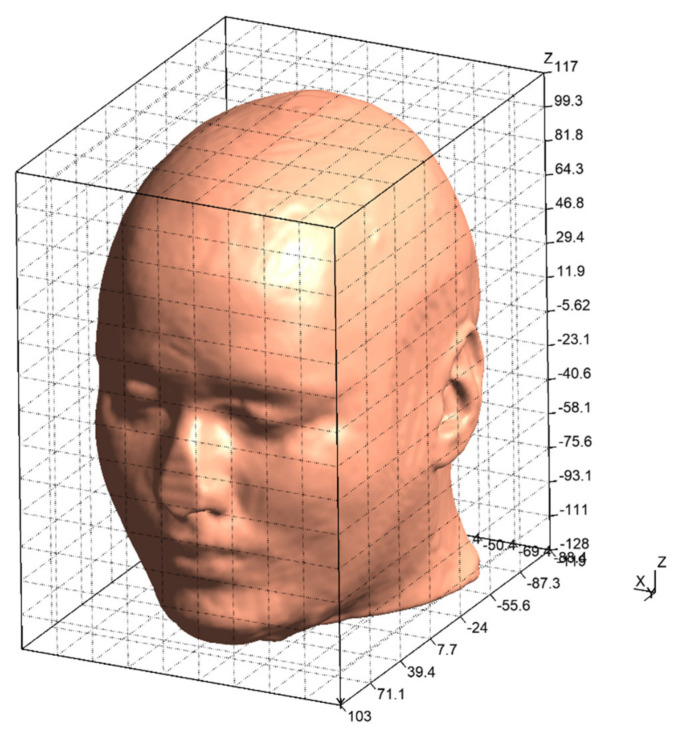
Full grid box of the mesh file of the human head model “Ernie”.

As mentioned above in Section 3, two steps are considered as the simulation in this study. Following the steps, two types of electrodes’ placements are prepared for the simulation. The first type is shown in Figure 2 where the electrodes are gradually separated away from each other. Basically, the electrode placement in Figure 2 is composed of a single pair of electrodes’ placements and two pairs of electrodes’ placements. Each of two pairs of electrode’s placements is a combination of two types of single pair of electrodes’ placements. Figure 3 illustrates the second type which is based on the electrode placement that stimulates the intended region on the eyeball surface [14]. The characteristics of each electrode placement shown in Figure 3 are described in Table 2. Table 3 and Table 4 show the detailed coordinates of each electrode placement used in the simulation. The coordinates in the tables represent the x-coordinate, y-coordinate, and z-coordinate from the left. The origin of the coordinate is set at the center of the human head model as shown in Figure 4. The size of the electrode was designed considering the gel pad of foc.us [29,30], which is the stimulator used in the previous study [13], and the size was 42 mm × 42 mm.

The electric stimulus value is based on 10 Hz and 1 mA referring to previous studies [13,14]. Besides, a simulation with 2 mA is also performed to verify the effect of electrical interference due to the magnitude of the current intensity. In this paper, the electric field value change due to the difference in the phase of the stimulation current is also verified when using multiple electrodes. When the forehead electrode is the anode, and the cheek electrode is the cathode, it is defined as the same phase condition. In the anti-phase condition, the cheek electrode is the anode, and the forehead electrode is the cathode. When two pairs of electrodes are used at the same time, in the case of the anti-phase condition, the cheek electrode on the right side of the face is the anode, and the forehead is the cathode, the electrode on the left cheek is the cathode, and the forehead is the anode. The phase difference verified by the simulation in this paper is 180°.

**Figure 5:**
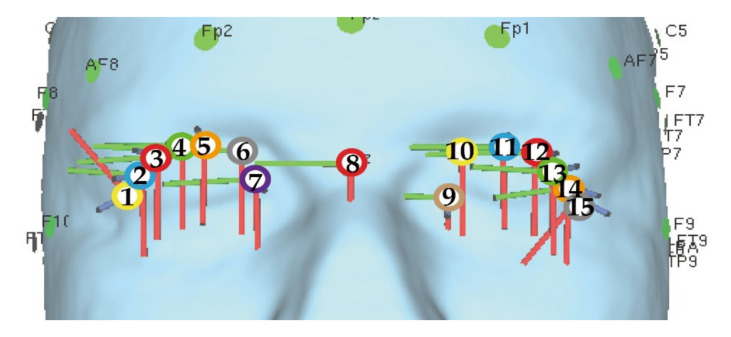
The points on the eyeball surface for observing the transition of the electric field value [14].

The points on the eyeball surface set for observing the transition of the electric field value are shown in Figure 5 and Table 5. Seven points were set on the surface of the right eye, seven points on the surface of the left eye, and one point on the upper part of the nose as a point between both eyes. In this paper, the transition of the electric field on the eyeball surface is simulated, and the norm value is used as the electric field value. The norm value of the electric field is the magnitude of the electric field vector and always takes a positive value. Furthermore, the information regarding the direction of the electric field is not included in the norm value.

### 3.2. Summarization of the Evaluation Method

**Figure 6:**
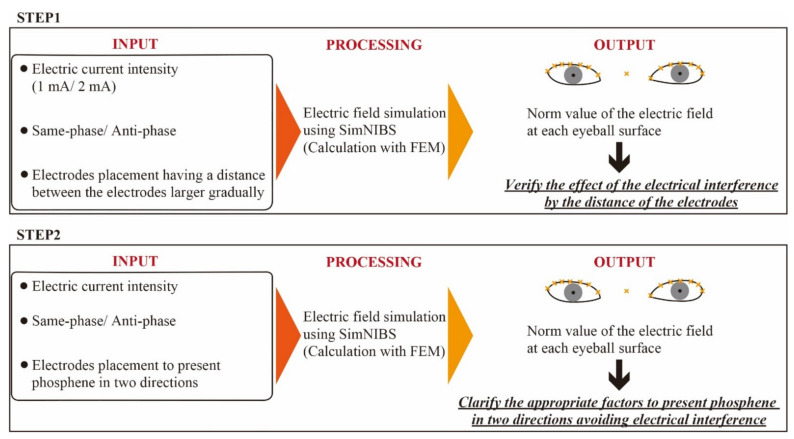
Diagram of the evaluation method for avoiding the electric interference while presenting phosphene in two directions.

This subsection describes the summarization of the evaluation method in this study. The diagram of the summarizing evaluation method mentioned in this section is shown in Figure 6. As mentioned at the beginning of this section, the evaluation is divided into two steps. The difference between the steps of the evaluation is the electrode placements in the input part. The appropriate factors (positional relation with electrodes, stimulation current intensity, and phase) will be clarified in the evaluation.

## 4. Evaluation

### 4.1. Electric Interference Due to the Distance of the Electrodes

**Figure 7:**
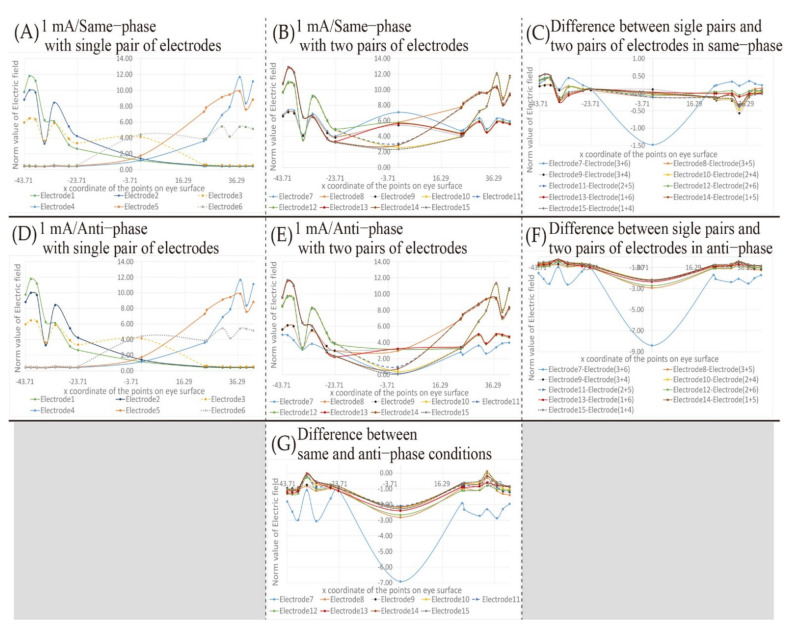
The transition of the electric field value on the eyeball surface with the electrode placement that is gradually separated from each other in 1 mA; (**A**) norm value of electric field in 1 mA and same-phase stimulus with single pair of electrodes; (**B**) norm value of electric field in 1 mA and same-phase stimulus with two pairs of electrodes; (**C**) difference of norm value of electric field in 1 mA and same-phase stimulus between single pair of electrodes and two pairs of electrodes conditions; (**D**) norm value of electric field in 1 mA and anti-phase stimulus with single pair of electrodes; (**E**) norm value of electric field in 1 mA and anti-phase stimulus with two pairs of electrodes; (**F**) difference of norm value of electric field in 1 mA and anti-phase stimulus between single pair of electrodes and two pairs of electrodes conditions; (**G**) difference of norm value of electric field in 1 mA with two pairs of electrodes between same-phase and anti-phase conditions.

Figure 7 shows the simulation result, changing the electric field on the eyeball surface under the stimulation condition of 1 mA using the first type of electrodes’ placement shown in Figure 2. Figure 7A,D shows the simulation results when a single pair of electrode placements are used. Figure 7B,E,G shows the simulation results when two pairs of electrode placements are used. Figure 7C shows the difference between the results of Figure 7A,B. Figure 7F shows the difference between the results of Figure 7D,E. Figure 7A–C shows the simulation results with the same phase stimulus, Figure 7D–F shows the simulation results with the anti-phase stimulus, and Figure 7G shows the difference between the results of Figure 7B,E. The horizontal axis of each simulation result stands for the x-coordinate of each point on the eyeball surface shown in Figure 5. Positive and negative values are the coordinates on the surface of the right eye and the left eye, respectively. The vertical axis stands for the norm value of the electric field, and the higher the value is, the stronger the influence of electrical stimulation is. Each point in Figure 7 represents the norm value of electric field at each point on the eyeball surface. The curve between each point in Figure 7 was obtained by line smoothing to connect between each measurement point. Based on the simulation result shown in Figure 7, the electric field value transition on the eyeball surface in each simulation condition is mentioned in this subsection.

Firstly, the electric field change on the eyeball surface during stimulation under the same phase condition shown in Figure 7A–C is focused on. In Figure 7A, it can be seen that the electrode placed on the right side of the face stimulates the surface of the right eyeball, and the one on the left side of the face stimulates the surface of the left eyeball. Since the electrode placement used in Figure 7B is composed of the combination of those used in Figure 7A, the peak transitions of the electric field values in Figure 7B are similar to the ones in Figure 7A. Figure 7C shows the numerical calculation of the difference between Figure 7A,B. Using electrode 7 in Figure 7 as an example, electrode 7 is composed of electrode 3 and electrode 6. Therefore, the result of electrode 7 in Figure 7C is obtained by subtracting the sum of the norm values of the electric fields of electrodes 3 and 6 from the norm value of the electric field of electrode 7. As a result, the norm value of the electric field in the center of the face of electrode 7 is remarkably low, and the difference in the electric field value on the eyeball surface of electrode 7 tends to be larger than the results of the other electrode placements. It is presumed that this is affected by electrical interference because the distance between the electrodes of electrode 7 is the shortest in the two pairs of electrode placements. On the other hand, it can be seen from Figure 7C that the values are generally within the range of ±0.50 except the one in the center of the face with electrode 7. In Figure 7A, the norm value of the electric field of the left eyeball is a constantly low value in the result of the electrode placement that stimulates the right eyeball such as electrode 4. Since the electric fields’ norm value on the unstimulated eyeball fluctuates within the range of 0–0.50, it is hard to consider that the value of 0.50 as the electric fields’ norm value has a significant influence as electrical interference. However, compared to the case using a single pair of electrodes’ placement alone, the norm value of the electric field with two pairs of electrode placements is larger at all coordinates. Hence, it is revealed that the electrical interference gives more potent stimulus.

Furthermore, the electric field change on the eyeball surface during stimulation under the anti-phase condition shown in Figure 7D–F is focused on. Figure 7D shows the results with a single pair of electrodes’ placements under anti-phase conditions. The result with the anti-phase shown in Figure 7D is ultimately the same waveform as the one with the same phase shown in Figure 7A. It is considered that this is because the norm value of the electric field was used for describing the simulation result, and there was no electrical interference from the other electrodes. The transition of the norm value of the electric field on the eyeball surface with two pairs of electrodes shown in Figure 7E does not seem to change from that under the same phase condition. However, the difference is remarkable in the center of the face. In Figure 7B, the norm value of the electric field in the center of the face shows a large convex waveform. In contrast, the norm value of the electric field in the center of the face in Figure 7E shows a significant depression under the anti-phase condition. Moreover, Figure 7F shows the numerical calculation of the difference between Figure 7D,E, and presents that all electrode placements and coordinates on the surface of the eyes indicate negative values. This means that under the anti-phase, the norm value of the electric field with a single pair of electrodes placement was larger than the one with two pairs of electrodes’ placement. The result was significantly different from the one of the stimulation conditions in the same phase. It can be considered that the stimuli were partially canceled by stimulating in the anti-phase.

Besides, Figure 7G shows the difference of the norm value of the electric field with two pairs of electrodes’ placements between the same phase stimulation conditions shown in Figure 7B and the anti-phase stimulation conditions shown in Figure 7E. Figure 7G shows that the electric field value at the coordinates near the center of the eyeball surface is larger than that in other regions. In addition, the norm value of the electric field near the center of the eyeball is smaller than that of other regions as seen from Figure 7A. Therefore, it is not easy to selectively stimulate the vicinity of the center of the eyeball. Since it is a region where stimulation is complex, it was difficult to be affected by phase interference. Besides, when using two pairs of electrode placements under the same phase stimulation conditions, the peaks in the region where current easily flows, such as the temporal side of the eye, were emphasized due to the electric interference. Therefore, when comparing the norm values of the electric field under the anti-phase and the same phase stimulation conditions, the difference between the value near the temporal eyeball and the one near the central eyeball surface appears prominently in comparison with Figure 7F.

**Figure 8:**
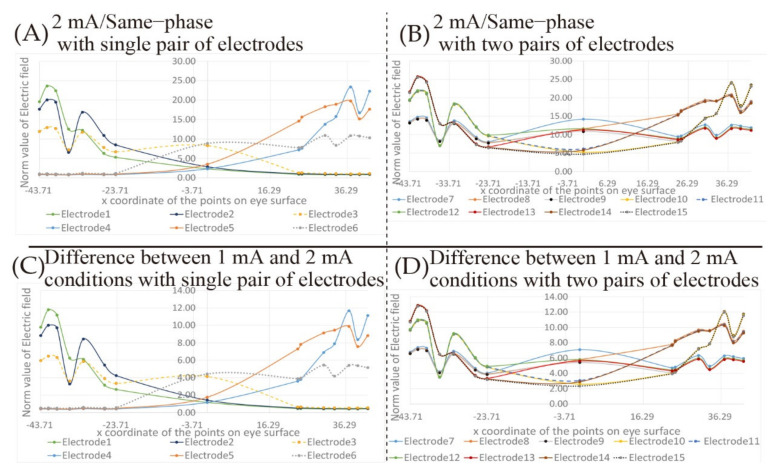
The transition of the electric field value on the eyeball surface with the electrode placement that is gradually separated from each other in 2 mA; (**A**) norm value of electric field in 2 mA and same-phase stimulus with single pair of electrodes; (**B**) norm value of electric field in 2 mA and same-phase stimulus with two pairs of electrodes; (**C**) difference of norm value of electric field in same-phase with single pair of electrodes between 1 and 2 mA conditions; (**D**) difference of norm value of electric field in same-phase with two pairs of electrodes between 1 and 2 mA conditions.

Furthermore, Figure 8 shows the simulation results changing the electric field on the eyeball surface under the same phase and 2 mA stimulation conditions using the electrodes’ placement shown in Figure 2. In Figure 8A,C, the simulation results are shown with a single pair of electrodes’ placement and Figure 8B,D shows the simulation results with two pairs of electrodes’ placements. Figure 8C,D shows the difference between the simulation results shown in Figure 8A,B and the simulation results of the 1 mA stimulus condition shown in Figure 5A and Figure 7B under the same phase condition.

Comparing Figure 7A with Figure 8A, it seems that the peak position of the electric field value does not change. The transition waveform also does not change, and the difference between Figure 7A and Figure 8A shown in Figure 8C is the same as Figure 7A. This tendency is the same as the result of the two pairs of electrodes’ placements in Figure 8D. Therefore, it can be seen that stimulation with 2 mA does not affect the waveform of the transition of the electric field value on the eyeball surface, and only the value with 2 mA is twice of the one with 1 mA.

From Figure 7 and Figure 8, the simulation results can be summarized as follows:

- When two pairs of electrodes’ placements are used under the same phase condition, the effect of electrical interference can be seen. However, the value does not fluctuate significantly within the region of the eyeball surface.
- When a single pair of electrodes is used under the anti-phase condition, the same result as the norm value of the electric field under the same phase condition is shown.
- When two pairs of electrodes’ placements are used under the anti-phase conditions, the electric field values near the center point between the electrodes are canceled out due to the electrical interference.
- The transition waveform and the peak position of the electric field are not changed due to the stimulation current intensity, which is irrespective of the electrode placements.
- The electric field value with 2 mA is twice that of the one with 1 mA.
- The coordinates of the peak position are not changed due to the influence of electrical interference, only the norm value of the electric field is changed.

### 4.2. The Effect of Electric Interference with Presenting the Phosphene at Two Directions

**Figure 9:**
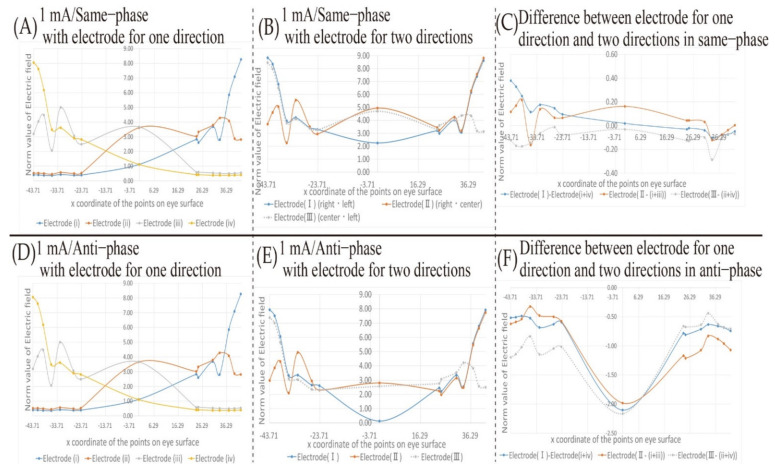
The transition of the electric field value on the eye surface with the electrode placement that stimulates the intended region on the eyeball surface in 1 mA; (**A**) norm value of electric field in 1 mA and same-phase stimulus with electrode to present phosphene in one direction; (**B**) norm value of electric field in 1 mA and same-phase stimulus with electrode to present phosphene in two directions; (**C**) difference of norm value of electric field in 1 mA and same-phase stimulus between electrode to present phosphene in one direction and electrode to present phosphene in two directions; (**D**) norm value of electric field in 1 mA and anti-phase stimulus with electrode to present phosphene in one direction; (**E**) norm value of electric field in 1 mA and anti-phase stimulus with electrode to present phosphene in two directions; (**F**) difference of norm value of electric field in 1 mA and anti-phase stimulus between electrode to present phosphene in one direction and electrode to present phosphene in two directions.

In this subsection, the simulation result of the electric field value on the eyeball surface using the electrode placements that stimulate intended regions on the eyeball surface is described. Figure 9 shows the simulation results of the electric field on the eyeball surface under the stimulation condition of 1 mA using the electrodes’ placements shown in Figure 3 when the phosphene is presented in two directions of the visual field. Figure 9A,D shows the simulation results of the electric field on the eyeball surface with four types of electrodes’ placements with the intention to present the phosphene in one direction, which is shown in Figure 3. Figure 9B,E shows the simulation results with two pairs of electrodes’ placements with the intention to present the phosphene in two directions, which is shown in Figure 3. Figure 9C shows the difference between the result of Figure 9A,B; and Figure 9F shows the difference between the result of Figure 9D,E. Figure 9A–C shows the simulation results of the same phase stimulus conditions. Figure 9D–F shows the simulation results of the anti-phase stimulus conditions.

Firstly, the simulation results under the same phase condition are focused on. In Figure 9A, it can be seen that each electrode stimulates the coordinates near the temporal side of both eyes and the third or fourth coordinates from the nasal side of the eyes. As shown in Figure 5, the third and fourth coordinates from the nasal side of the eyes are the regions near the center of the eyeball. Therefore, it is considered that the electrode placements in Figure 9A can selectively stimulate the vicinity of the temporal part of the eyes and the vicinity of the center of the eyeball. Furthermore, the result of Figure 9B is focused on. Electrodes (I) are electrodes’ placements that present phosphenes on the right and left sides of the visual field, and Figure 9B shows that peaks stand at both temporal sides of the eye safely. Electrode (II) is an electrodes’ placement that presents phosphenes on the right side and in the center of the visual field. In Figure 9B, peaks appear at the coordinates near the temporal side of the right eyeball and near the center of the left eyeball. Electrode (III) is an electrodes’ placement that presents phosphenes in the center and left side of the visual field. In Figure 9B, peaks appear at the coordinates near the temporal side of the left eyeball and near the center of the right eyeball. The norm value of the electric field on the eyeball surface shown in Figure 9C is the difference between the result of Figure 9A,B. Compared with the case of Figure 7C, the difference is within a smaller range. Therefore, it is considered that the influence of electrical interference is more suppressed than using the electrode placements shown in Figure 2.

Secondly, the simulation results under anti-phase conditions are focused on. Since Figure 9D is the simulation result with a single pair of electrodes’ placement, the simulation result is the same as in Figure 7D under the same phase condition. Moreover, in Figure 9E, it can be seen that the peak stands at almost the same position as Figure 9B. Besides, as seen in Figure 7E, it can be seen that the norm value of the electric field at the coordinates of the center of the face has dropped in Figure 9E as well. Furthermore, focusing on the peak at the coordinates near the center of the right eyeball with electrode (III), the electric field value in anti-phase is smaller than the value in other regions. It is considered that this change in the peak value is also due to the cancellation of the norm value of the electric field near the nasal side of the right eyeball each other by stimulation in the anti-phase. Figure 9F shows the difference between Figure 9D,E. Similar to Figure 7F, negative values are shown at all coordinates, and cancellation due to electrical interference can be seen in Figure 9F. Compared with the two pairs of electrodes’ placements in Figure 2, the electrode placements in Figure 3 are farther apart. However, the effect of the cancellation of electric field values due to anti-phase conditions should be considered.

## 5. Discussion

In Section 4, the influence of the electrical interference on the electric field value on the eyeball surface was shown under various stimulus conditions. Thereby, in this section, the important points for presenting a phosphene in two directions of the visual field are presented. Besides, a method for correctly presenting a phosphene in two directions in a walking support system for the visually impaired is discussed.

From Figure 5, Figure 6 and Figure 7, it is clear that the peak position of the electric field value on the eyeball surface does not change due to the electrical interference when two pairs of electrodes’ placements are used. On the other hand, the magnitude of the norm value of the electric field is considered to be one of the causes of the change in the presentation position of the phosphene.

Figure 7C shows that the amplitude of the electric field value on the eyeball surface is positive at almost all coordinates on the eyeball surface. Therefore, using two pairs of electrodes’ placements may stimulate the eye surface with a higher electric field norm value than using a single pair of electrodes placement. The threshold value of the norm value of the electric field on the eyeball surface required for the presentation of the phosphene has not been clarified as a concrete numerical value. However, when plural electrodes are used simultaneously, the threshold value is exceeded, and a phosphene is perceived in a region showing a relatively small peak of the electric field. Furthermore, the threshold value of the electric fields’ norm value on the eyeball surface required for the presentation of the phosphene needs to be considered even in the situation using a single pair of electrodes’ placement. By selecting 2 mA as the stimulus current intensity, as shown in Figure 8, the norm value of the electric field, which is twice as high as that when stimulating at 1 mA, was presented. This change of the norm value of the electric field implies that a phosphene is presented at an unintended position. For example, comparing the transition of the electric field norm value of electrode 3 in Figure 7A and Figure 8A, the norm of the electric field with 1 mA in the region around the nasal side of the left eyeball, which was relatively smaller than the norm of the electric field with 2 mA. It can be seen that when the electric current intensity is 2 mA, the nasal part of the value of electrode 3 is close to the norm value of the peak electric field using 1 mA. In other words, if the magnitude of the norm value of the electric field can be used as threshold value in the perception of a phosphene, the intensity of the light of the phosphene with 2 mA will be observed over the entire field of human vision which is controlled by the left eyeball. Therefore, the selection of the stimulus value is important for the accurate presentation of the phosphene. It is necessary to clarify the numerical relation between the electric field value on the eyeball surface and the observed phosphene by experiments.

Focusing on the electrical interference with the electric field on the eyeball surface under anti-phase stimulation, there are significant characteristics of the electric field value shown in Figure 7 and Figure 9. Figure 5 and Figure 7 show that the electrical interference caused by stimulation with anti-phase when using the two pairs of electrodes’ placements has different characteristics from the same phase stimulation result. The norm value of the electric field was lower than that under the stimulation conditions in the same phase. This is because of the cancellation caused by the currents with anti-phase. In Figure 7, some norm values of the electric field at the center of the face were found to be significantly depressed. Considering the positional relation of each of the two pairs of electrodes placements, it can be seen that the electric field value near the center point between the electrodes on the forehead tends to be low. As a prominent example, the electrodes’ placements in which the norm value of the electric field near the center of the forehead is largely canceled are electrodes 7, 10, 11, 14, and 15. Since these electrodes have a center point between the electrodes near the center of the forehead, it is considered that the significant depression of the electric field at the center of the face was obtained. Therefore, the canceled coordinates of the electric field can be predicted by the positional relation between the electrodes.

Finally, the method of presenting phosphene in two directions is mentioned, which is important in this study. It has been verified that phosphene can be presented in the “right/left direction of the human vision”, “right/center direction of the human vision”, and “center/left direction of the human vision” using the three electrode placements. As a result, it was shown in Figure 9 that the peak is generated at the coordinates on the corresponding eyeball surface without any problem.

However, there are still issues to be solved in the presentation of the phosphene toward the center. As mentioned above, the presentation of the phosphene is related to the magnitude of the norm value of the electric field. However, the value of the peak appearing at the 3rd and 4th coordinates from the nasal side of the eye on the eyeball surface, which is currently related to the presentation toward the central vision, is smaller than the value of the peak appearing near the temporal eyeball of both eyes. Moreover, using two pairs of electrodes’ placements simultaneously, the value of a relatively small peak standing at another coordinate becomes large, and a phosphene might be presented at an unintended position. Therefore, when presenting the phosphene in two directions, it is necessary to stimulate only the target region by selecting the stimulation current intensity and the phase to reliably present the phosphene in the central direction.

In the verification for presenting the phosphene in two directions under the anti-phase stimulus shown in Figure 9E, it was shown that the norm value of the electric field near the nasal side of the eye is relatively small. When the area around the nasal side of the eye is electrically stimulated, a phosphene is presented from the center to the left side of the visual field. This is because of an anatomical positional relation when the right eyeball is stimulated. Therefore, when presenting a phosphene over the entire visual field, a method of stimulating the vicinity of the nasal eyeball of both eyes can be considered. However, when presenting the phosphene in two directions of the visual field, it is considered that avoiding the stimulation near the nasal side of the eyeball of both eyes is needed for presenting the phosphene more selectively. Therefore, when presenting a phosphene in two directions, it is considered to be an effective means to select anti-phase stimuli conditions.

Besides, the following two measures can be taken to improve the presentation of the phosphene in the central direction.

- Stimulate only the right eyeball when presenting the phosphene in the “right/center direction” and stimulate only the left eyeball when presenting the phosphene in the “center/left direction.”
- Set the stimulation current intensity of the electrode that presents the phosphene toward the center of the visual field higher than the other electrodes.

**Figure 10:**
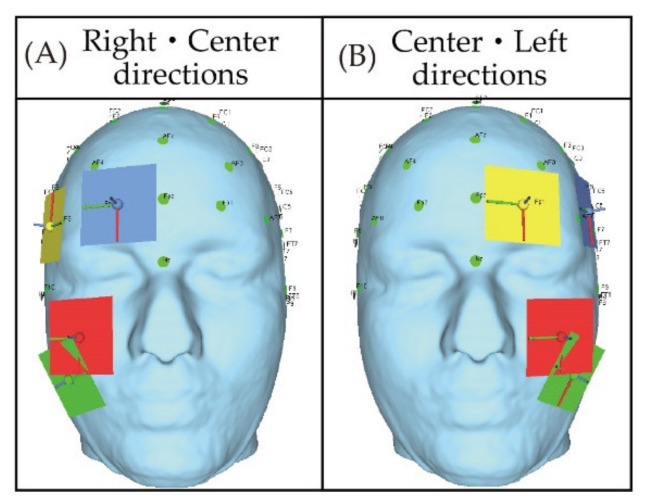
The electrode placement that stimulates only the right eyeball and the electrode placement that stimulates only the left eyeball.

**Figure 11:**
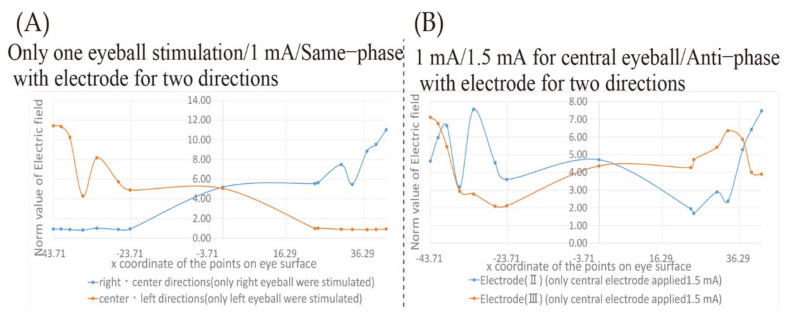
The transition of the electric field value on the eye surface with the electrode placement that presents the phosphene in two directions of visual field; (**A**) norm value of electric field in 1 mA and same-phase stimulus with electrode to present phosphene in two directions that stimulate only one eyeball; (**B**) norm value of electric field in anti-phase stimulus with electrode to present phosphene in two directions that stimulate with 1 mA for the nasal eyeball and 1.5 mA for the central eyeball.

Firstly, the first measure will be described. In the electrode placement used in Figure 9, in the case of presentation in the “right/center direction,” the vicinity of the temporal side of the right eyeball and the vicinity of the center of the left eyeball were stimulated. In the case of presentation in the “center/left direction,” the vicinity of the center of the right eyeball and the vicinity of temporal side of the left eyeball were stimulated. However, by using two pairs of electrodes’ placements, the norm value of the electric field of the second peak takes a large value, and the phosphene might be perceived to the left, which should be presented in the central direction in the “right/central direction.” Therefore, it is considered that the erroneous presentation of the phosphene can be avoided by fixing the eyeball to be stimulated in advance. Figure 10 shows the electrodes’ placements in which the stimulated eyeball is fixed, and the phosphene is presented in the “right/center direction” and the “center/left direction”. Besides, Figure 11A shows the transition of the electric field on the eyeball surface with the electrodes’ placements shown in Figure 10 in same-phase stimulus. Accordingly, there are no peaks at coordinates other than the stimulated region. Therefore, it is considered that this means that the presentation of the phosphene to an unintended region can be avoided.

Secondly, the second measure will be described. Electrodes (II) and electrodes (III) shown in Figure 3 are used in the second measure, and only the stimulation current intensity from the electrode that stimulates the vicinity of the center of the eyeball surface is set to 1.5 mA, and then the stimulation conditions in anti-phase were used for verification. Figure 11B shows the norm value of the electric field on the eyeball surface with that stimulation condition. Compared with the result shown in Figure 9E, it can be seen that the value of the peak at the third and fourth coordinates from the nasal side of the eye is close to those of the peak at the temporal side of the eye. In addition, by applying the anti-phase stimulus, the norm values of the electric field at the coordinates from the nasal side of the eye to the center of the face are close to those shown in Figure 9B with 1 mA, even though the stimulus current intensity is set as high as 1.5 mA. Therefore, it is considered that sufficient electrical stimuli can be given to the target region by applying the stimulus to the central eyeball surface at a higher current intensity than the other electrodes under the anti-phase stimulus.

## 6. Conclusions

In this paper, the electrode placements and stimulation factors that enable the presentation of phosphene in two directions of the visual field were examined. First, focusing on the electric field on the eyeball surface induced by using two pairs of electrode placements, the effect of electrical interference on the norm value was investigated. It was clarified that although the magnitude of the norm value of the electric field changes, the peak of the coordinates does not change. Second, how to present the phosphene only in the intended two directions of the visual field using the specified electrode placements avoiding electrical interference was clarified. As a result, it was shown that the following methods in Table 6 are effective.

The relation between the electric field on the eyeball surface and each factor (positional relation with electrodes, stimulation current intensity and phase) was clarified. Thereby, a method for presenting phosphenes at the intended position was established. In this paper, the phosphene presentation in three or more directions of the visual field was not focused on. However, even when the phosphene should be presented in a lot of positions in the visual field, it is possible to determine the stimulation method in the same way as mentioned above.

In this paper, the phosphene presentation with stimulating eyeball was focused on. However, depending on particular situation, some visually impaired people cannot perceive the phosphene. Since the eyeball is the peripheral part in the visual pathway, the visually impaired people who have damage at the optic nerve or visual cortex cannot process the electric stimulus to the eye. Therefore, how to control the phosphene presentation by stimulating the human brain should be investigated in the future. According to [11], it was mentioned that the phosphene is derived from the eyeball even though the electric stimulus is applied to the visual cortex. It is necessary to show that presenting phosphene derived from the visual cortex is possible by stimulating the human brain. This is because the phosphene can probably be presented to a visually impaired person with optic nerve damage if the phosphene presentation is not solely due to the retina. There are two ways to clarify these issues. One is to conduct a verification with visually impaired people who have either optic nerve blockage or aneurysms [31] as subjects. The other is to establish an electrode placement by which the current does not flow into the eyeball direction by simulation and perform the verification experiment based on the electrode placement. The former can certainly show that the phosphene is obtained by stimulation of the visual cortex. However, it is difficult to ask subjects for cooperation. The latter has an advantage of less burden to subjects since it is possible to decrease the number of experiments by simulating the appropriate electrode placement before the subject evaluation. However, it is also necessary to confirm whether the current path follows the results shown by simulation in this paper.

## Figures and Tables

**Table 1:** The electrical conductivity for 10 Hz alternating current stimulation [27,28].

Tissues	Electrical Conductivity
White matter	0.027656
Gray Matter	0.027512
CSF ^1^	2.0000
Bone	0.020028
Head skin	2.0000 × $10^{-4}$
Eye ball	0.41113

^1^ CSF (cerebral spinal fluid).

**Table 2:** The potential location of the phosphene in each electrode used in the second simulation.

Electrode Placement	The Potential Position of the Phosphene in the Visual Field
i	Right hand of the visual field
ii	Central visual field
iii	Central visual field
iv	Left hand of the visual field
I	Right and left hand of the visual field
II	Right and center of the visual field
III	Center and left of the visual field

**Table 3:** The coordinates of each electrode placement used in the first simulation.

Electrode Placement	Coordinate of the Electrode on the Forehead	Coordinate of the Electrode on the Cheek	Coordinate of the Electrode on the Other Forehead	Coordinate of the Electrode on the Other Cheek
1	(−56.65, 68.06, 31.40)	(−45.91, 77.21, −35.86)	-	-
2	(−41.17, 81.53, 31.84)	(−45.91, 77.21, −35.86)	-	-
3	(−20.83, 88.39, 35.82)	(−45.91, 77.21, −35.86)	-	-
4	(55.27, 69.06, 27.18)	(44.43, 76.69, −36.22)	-	-
5	(37.57, 83.32, 30.48)	(44.43, 76.69, −36.22)	-	-
6	(19.97, 89.36, 35.38)	(44.43, 76.69, −36.22)	-	-
7	(19.97, 89.36, 35.38)	(44.43, 76.69, −36.22)	(−20.83, 88.39, 35.82)	(−45.91, 77.21, −35.86)
8	(37.57, 83.32, 30.48)	(44.43, 76.69, −36.22)	(−20.83, 88.39, 35.82)	(−45.91, 77.21, −35.86)
9	(55.27, 69.06, 27.18)	(44.43, 76.69, −36.22)	(−20.83, 88.39, 35.82)	(−45.91, 77.21, −35.86)
10	(55.27, 69.06, 27.18)	(44.43, 76.69, −36.22)	(−41.17, 81.53, 31.84)	(−45.91, 77.21, −35.86)
11	(37.57, 83.32, 30.48)	(44.43, 76.69, −36.22)	(−41.17, 81.53, 31.84)	(−45.91, 77.21, −35.86)
12	(19.97, 89.36, 35.38)	(44.43, 76.69, −36.22)	(−41.17, 81.53, 31.84)	(−45.91, 77.21, −35.86)
13	(19.97, 89.36, 35.38)	(44.43, 76.69, −36.22)	(−56.65, 68.06, 31.40)	(−45.91, 77.21, −35.86)
14	(37.57, 83.32, 30.48)	(44.43, 76.69, −36.22)	(−56.65, 68.06, 31.40)	(−45.91, 77.21, −35.86)
15	(55.27, 69.06, 27.18)	(44.43, 76.69, −36.22)	(−56.65, 68.06, 31.40)	(−45.91, 77.21, −35.86)

**Table 4:** The coordinates of each electrode placement used in the second simulation [14].

Electrode Placement	Coordinate of the Electrode on the Forehead	Coordinate of the Electrode on the Cheek	Coordinate of the Electrode on the Other Forehead	Coordinate of the Electrode on the Other Cheek
i	(62.68, 57.05, 27.99)	(44.43, 76.69, −36.22)	-	-
ii	(23.16, 88.73, 34.62)	(51.05, 66.06, −59.22)	-	-
iii	(−24.47, 88.32, 33.67)	(−53.77, 65.46, −56.93)	-	-
iv	(−64.06, 54.13, 28.95)	(−45.91, 77.21, −35.86)	-	-
I	(62.68, 57.05, 27.99)	(44.43, 76.69, −36.22)	(−64.06, 54.13, 28.95)	(−45.91, 77.21, −35.86)
II	(62.68, 57.05, 27.99)	(44.43, 76.69, −36.22)	(−24.47, 88.32, 33.67)	(−53.77, 65.46, −56.93)
III	(23.16, 88.73, 34.62)	(51.05, 66.06, −59.22)	(−64.06, 54.13, 28.95)	(−45.91, 77.21, −35.86)

**Table 5:** The coordinate of each point on the eyeball surface for observing the transition of the electric field value [14].

Point on the Eyeball Surface	Coordinate of Each Point
Point 1	(42.09, 67.86, 3.46)
Point 2	(39.46, 69.97, 4.62)
Point 3	(37.15, 70.94, 7.02)
Point 4	(33.36, 70.69, 10.94)
Point 5	(30.51, 71.00, 6.57)
Point 6	(24.51, 70.50, 7.92)
Point 7	(23.71, 70.62, 6.51)
Point 8	(−0.05, 87.00, 4.83)
Point 9	(−23.92, 67.85, 3.6)
Point 10	(−26.97, 69.96, 6.29)
Point 11	(−32.52, 71.87, 8.17)
Point 12	(−36.13, 70.88, 10.3)
Point 13	(−39.46, 71.41, 3.32)
Point 14	(−41.73, 70.19, 2.63)
Point 15	(−43.71, 67.36, 2.24)

**Table 6:** The methods to present the phosphene in two directions in the visual field.

Phosphene Presentation Position	Appropriate Electrode Placement	Current Intensity	Phase Condition
right/left direction	Electrode (I), which stimulates temporal side of both eyeballs	1 mA	Anti-phase
right/center direction	The electrode placements which stimulate only the right eyeball	1 mA	Same-phase
	The electrode placements which stimulate the temporal side of the right eyeball and the vicinity of the center of the left eyeball surface	Vicinity of the center: 1.5 mA Temporal side: 1 mA	Anti-phase
center/left direction	The electrode placements which stimulate only the left eyeball	1 mA	Same-phase
	The electrode placements which stimulate the temporal side of the left eyeball and the vicinity of the center of the right eyeball surface	Vicinity of the center: 1.5 mA Temporal side: 1 mA	Anti-phase
